# Challenges recruiting to a proof-of-concept pharmaceutical trial for a rare disease: the trigeminal neuralgia experience

**DOI:** 10.1186/s13063-018-3045-1

**Published:** 2018-12-27

**Authors:** Joanna M. Zakrzewska, Joanne Palmer, Lars Bendtsen, Giulia Di Stefano, Dominik A. Ettlin, Stine Maarbjerg, Mark Obermann, Valerie Morisset, Deb Steiner, Simon Tate, Giorgio Cruccu

**Affiliations:** 1 Facial Pain Unit, Division of Diagnostic, Surgical and Medical Sciences, Eastman Dental Hospital, University College London Hospitals NHS Foundation Trust/University College London, London, UK; 2 Convergence Pharmaceuticals Ltd, Cambridge, UK; 3 Danish Headache Center, Department of Neurology, Rigshospitalet—Glostrup, University of Copenhagen, Valdemar Hansens Vej 5, 2600 Glostrup, Denmark; 4 Department of Human Neuroscience, Sapienza University of Rome, Rome, Italy; 5 Interdisciplinary Orofacial Pain Unit, Clinic of Masticatory Disorders, Center of Dental Medicine, University of Zurich, Zurich, Switzerland; 6 Department of Neurology and German Headache Center, University of Duisburg-Essen, Essen, Germany; 7 Center for Neurology, Asklepios Hospitals Schildautal, Seesen, Germany; 8 Biogen, Cambridge, MA USA; 9 Eastman Dental Hospital, 256 Gray’s Inn Road, London, UK; 10 Pain Management Centre, University College London Hospitals NHS Foundation Trust, London, UK

**Keywords:** Clinical trial, Vixotrigine, Sodium channel blocker, Underrepresentation

## Abstract

**Background:**

This study aimed to describe recruitment challenges encountered during a phase IIa study of vixotrigine, a state and use-dependent Nav1.7 channel blocker, in individuals with trigeminal neuralgia.

**Methods:**

This was an international, multicenter, placebo-controlled, randomized withdrawal study that included a 7-day run-in period, a 21-day open-label phase, and a 28-day double-blind phase in which patients (planned *n* = 30) were randomized to vixotrigine or placebo. Before recruitment, all antiepileptic drugs had to be stopped, except for gabapentin or pregabalin. After the trial, patients returned to their original medications. Patient recruitment was expanded beyond the original five planned (core) centers in order to meet target enrollment (total recruiting sites *N* = 25). Core sites contributed data related to patient identification for study participation (prescreening data). Data related to screening failures and study withdrawal were also analyzed using descriptive statistics.

**Results:**

Approximately half (322/636; 50.6%) of the patients who were prescreened at core sites were considered eligible for the study and 56/322 (17.4%) were screened. Of those considered eligible, 26/322 (8.1%) enrolled in the study and 6/322 (1.9%) completed the study. In total, 125 patients were screened across all study sites and 67/125 (53.6%) were enrolled. At prescreening, reasons for noneligibility varied by site and were most commonly diagnosis change (78/314; 24.8%), age > 80 years (75/314; 23.9%), language/distance/mobility (61/314; 19.4%), and noncardiac medical problems (53/314; 16.9%). At screening, frequently cited reasons for noneligibility included failure based on electrocardiogram, insufficient pain, and diagnosis change.

**Conclusions:**

Factors contributing to recruitment challenges encountered in this study included diagnosis changes, anxiety over treatment changes, and issues relating to distance, language, and mobility. Wherever possible, future studies should be designed to address these challenges.

**Trial registration:**

ClinicalTrials.gov, NCT01540630.

EudraCT, 2010-023963-16. 07 Aug 2015.

## Background

Randomized controlled trials underpin the development of new drugs and therapies, but challenges with patient recruitment are often encountered, delaying study completion. A review of 114 multicenter trials conducted by two UK funding bodies found that fewer than one-third of trials recruited to their original target within the specified timeframe [1]. Recruitment challenges can be even tougher when the target population includes patients with a rare disease [2]. In addition, studies have found that elderly patients and those from racial and ethnic minorities were underrepresented in pivotal US trials from 2011 to 2013 [3]. It is recognized, however, that the inclusion of elderly patients, often unqualified to participate in clinical trials because of comorbid conditions [4], might help to uncover drug–drug or drug–disease interactions. Failure to achieve the required sample size affects the reliability and generalizability of the results.

While there are varied barriers to trial recruitment, studies have shown that the potential to receive placebo therapy is of particular concern to patients [5–8]. Other barriers identified in the literature include trial demands (procedures/appointments that may cause discomfort or inconvenience), travel time and travel costs, uncertainty around effects of treatment or unwillingness to change medication, and random allocation to study arms [7, 8]. Clinical trials involving therapies for rare diseases require involvement of multiple sites, usually with relatively few participants from each site, in order to enroll the target number of participants [2]. Thus, clinical trials that include patients with a rare disease present an even greater challenge to physicians, most notably because of the small numbers of eligible patients and limited treatment options that influence patient reluctance to receive placebo [5, 9].

Trigeminal neuralgia (TN) is characterized by recurrent paroxysms of pain that last from seconds to 2 min and are triggered by seemingly harmless sensory stimuli [10]. The pain is best described as like an electric shock, is of severe intensity, and has a unilateral distribution that does not extend beyond the trigeminal nerve distribution. TN is considered rare, with incidence in the range of 4.2–28.9 per 100,000 persons [11–13]. In addition to the challenges inherent in a rare condition, there are other unique features of TN that may be associated with further challenges to recruitment. For example, TN is characterized by very severe pain as well as unpredictable remission and relapse periods, which may increase uncertainty around trialing new therapies, result in variability, and lead to concerns about receiving placebo treatment. Incidence of TN also increases with age, with the peak age of onset around age 50–60 years [14], so the population is skewed toward a harder-to-recruit older population.

Vixotrigine is a state- and use-dependent inhibitor of Nav1.7 voltage-gated sodium channels. We recently reported a phase IIa trial (ClinicalTrials.gov NCT01540630 and EudraCT 2010-023963-16) in individuals with TN that used a randomized withdrawal design to evaluate the efficacy and safety of vixotrigine treatment [15]. The randomized withdrawal design was selected to address concerns around placebo therapy by minimizing the time patients would spend on placebo in the absence of effect. Nonetheless, while the trial recruited the required number of patients, additional centers were required and the study ran over a longer timeframe than originally anticipated. We conducted an analysis of the reasons for recruitment difficulties experienced in the phase IIa study in order to improve understanding of the challenges in TN trial recruitment and gather learnings for future trial design.

## Methods

The results from the vixotrigine phase IIa study and the trial design have been published previously [15, 16]. In brief, the phase IIa study was an international, double-blind, multicenter, placebo-controlled, randomized withdrawal study involving 25 secondary/tertiary care centers in 12 countries (United Kingdom, Germany, Denmark, Italy, Switzerland, South Africa, France, Spain, Estonia, Latvia, Lithuania, and Romania), conducted from April 2012 to February 2014. The study comprised a 7-day run-in period, a 21-day open-label phase during which all patients received open-label vixotrigine, and a 28-day double-blind phase in which patients were randomized to vixotrigine or placebo. Patients meeting defined response criteria during the open-label phase were eligible for inclusion in the double-blind phase [15, 16]. At the end of the study, patients went back to their original medications.

### Phase IIa study patient population and target recruitment

Prescreening criteria were determined by the site and were dependent upon the information available to the site regarding individual patients. Sites identified patients via study center databases, advertising through patient support groups, or recruiting directly from specialized headache and facial pain clinics. Identified patients underwent protocol-defined screening (history and physical, 12-lead electrocardiogram (ECG), clinical laboratory) before study enrollment. The diagnosis of TN was confirmed by a data monitoring committee, comprised of three lead investigators, before patients were formally enrolled. Included patients were aged 18–80 years (increased from 70 years in a protocol amendment, May–August 2012) with active TN. Diagnosis was based on the second edition of the International Classification of Headache Disorders [17, 18]. Symptomatic (secondary) TN was excluded by imaging. Patients were required to have at least moderate pain and frequent daily paroxysms of pain, defined as at least three paroxysms per day, each rated at intensity ≥ 4 on a pain intensity numerical rating scale, on ≥ 4 days during the 7 days before study entry. Known nonresponders to sodium channel blockers at therapeutic doses were excluded.

This was a phase IIa study and, like most drugs at this stage, had limited available safety data. As in many early-phase studies, this knowledge void resulted in extensive exclusion criteria. Several other factors related to the limited exposure of the drug to patients resulted in further exclusion criteria: uncontrolled or poorly controlled hypertension; significant cardiovascular, gastrointestinal, or renal disease; or other conditions known to interfere with the absorption, distribution, metabolism, or excretion of drugs. Fridericia’s formula had to be < 450 ms in two of three ECGs done at screening. The use of a wide range of concomitant drugs had to be excluded and the only anticonvulsants that were allowed were gabapentin or pregabalin. Patients were not permitted to use other sodium channel blockers (including carbamazepine and oxcarbazepine) during the study. Further details on inclusion/exclusion criteria are available in prior publications [15, 16].

The sample size calculation estimated that up to 70 participants were needed for the open-label phase in order for 30 patients to enter the randomized double-blind phase and provide sufficient statistical power for the primary study endpoint (reduction in treatment failure with vixotrigine vs placebo throughout the double-blind phase) [16].

### Analysis of challenges in recruitment and retention

The current analysis explored the challenges faced in recruiting participants to the phase IIa study, using prescreening and screening data from the five original (core) sites that maintained a complete record of the reasons why patients were not recruited into the trial. Prescreening/screening data were not formally databased by the study sponsor, but were captured in site records. The five core sites were Essen (Germany), Glostrup (Denmark), London (United Kingdom), Rome (Italy), and Zurich (Switzerland). Because TN frequently presents in the lower part of the face, patients are often initially seen by dentists who are skilled at diagnosis of TN. In Zurich, patients are referred specifically to an oral medicine unit within a university dental school rather than a neurology department. The other sites were specific headache or facial pain units or neuropathic pain centers, run principally by neurologists. Prescreening at these sites was based on analysis of database records and clinic visits.

We also analyzed data on patient withdrawals during the open-label period of the phase IIa study, based on the full study population (25 centers) [16], in order to further understand the factors influencing trial retention.

## Results

### Prescreening, screening, and enrollment at five core sites

As previously reported, 125 patients were screened; 53.6% (67/125) patients entered the open-label phase, and 23.2% (29/125) patients were treated in the double-blind phase [16]. Analyzing data from the five core sites only, a total of 636 patients were included in prescreening, of whom 322 (50.6%) were considered eligible for screening per study criteria and 314 (49.4%) were noneligible. Patients with low or no pain at the time of prescreening may have been considered eligible because their status could change over time. Of the eligible patients, 56/322 (17.4%) were ultimately screened and 26/322 (8.1%) were enrolled (of these, 15/322 (4.7%) proceeded to the randomized double-blind phase). The core group enrolled 15/29 (51.7%) of the double-blind population.

Table 1 presents the breakdown of eligible, noneligible, screened, and enrolled patients by study site. By site, the proportion of noneligible patients ranged from 11.1% (Zurich) to 72.4% (Glostrup). The proportion proceeding to screening ranged from 1.3% (London) to 62.5% (Zurich), and those proceeding to enrollment from 0% (London) to 18.8% (Zurich).

**Table 1:** Eligible/noneligible patients at prescreening and numbers screened and enrolled for the five core sites

Study site	Total pool	Noneligible				Eligible				Screened	Enrolled
		*n* (% of total pool)	Mean age (years)	Male, *n* (% of noneligible)	Female, *n* (% of noneligible)	*n* (% of total pool)	Mean age (years)	Male, *n* (% of eligible)	Female, *n* (% of eligible)	*n* (% of eligible)	*n* (% of eligible)
Essen	109	24 (22.0)	71	7 (29.2)	17 (70.8)	85 (78.0)				5 (5.9)	2 (2.4)
Glostrup	221	160 (72.4)	67	54 (33.8)	106 (66.2)	61 (27.6)	64	29 (47.5)	32 (52.5)	12 (19.7)	6 (9.8)
London	161	82 (50.9)	67	29 (35.4)	53 (64.6)	79 (49.1)	60	30 (38.0)	49 (62.0)	1 (1.3)	0
Rome	130	46 (35.4)	69	14 (30.4)	32 (69.6)	84 (64.6)	63	28 (33.3)	56 (66.7)	28 (33.3)	15 (17.9)
Zurich	15	2 (11.1)	62	0	2 (100.0)	13 (88.9)	58	4 (30.8)	9 (69.2)	10 (62.5)	3 (18.8)
TOTAL	636	314 (49.1)				322 (50.6)				56 (17.4)	26 (8.1)

The mean age of eligible patients tended to be lower than for noneligible patients. The proportion of male patients was slightly higher in the eligible group versus the noneligible group.

### Reasons for noneligibility at prescreening and screening

**Fig. 1:**
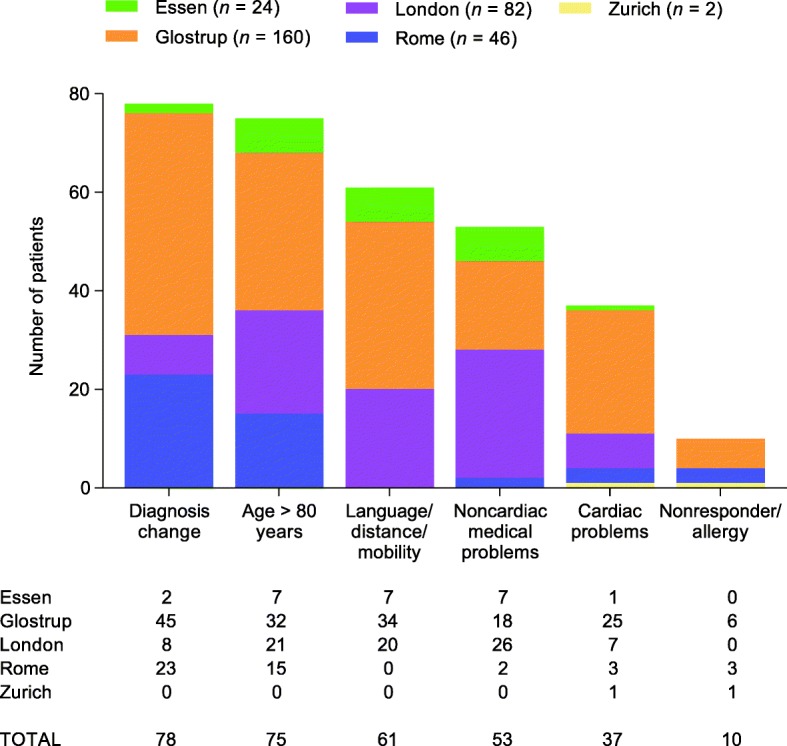
Reasons for prescreening failures for the five core sites. Diagnosis change = initial diagnosis of classical trigeminal neuralgia (TN), but later recorded as not classical trigeminal neuralgia (TN). Age > 80 years = noneligible because of age over the upper limit of 80 years per trial inclusion criteria. Language/distance/mobility = unable to participate in trial due to language barriers, distance from trial sites, or limited mobility prohibiting attendance at required study visits. Noncardiac medical problems = noneligible because of noncardiac medical issues prohibited under trial entry criteria. Cardiac problems = noneligible because of cardiac medical issues prohibited under trial entry criteria, including Fridericia’s formula requirements (< 450 ms in two of three electrocardiograms done at screening). Nonresponder/allergy = noneligible because known nonresponders to sodium channel blockers at therapeutic doses, or due to a history of hypersensitivity, or due to a history of drug or other allergy that contraindicates their participation

Figure 1 illustrates the reasons documented for noneligibility at prescreening at the five core sites. The most common reason for noneligibility at prescreening was diagnosis change (78/314; 24.8%), followed by age > 80 years (75/314; 23.9%), language/distance/mobility (61/314; 19.4%), and noncardiac medical problems (53/314; 16.9%), although the relative frequency varied by site (Fig. 1). Diagnosis change was the most common reason in Glostrup and Rome, while noncardiac medical problems was the most common reason in London. At the Essen site, noncardiac medical problems, age > 80 years, and language/distance/mobility were the most frequent reasons. At the Zurich site, only two patients were considered noneligible (due to noncardiac medical problems and nonresponder/allergy).

**Fig. 2:**
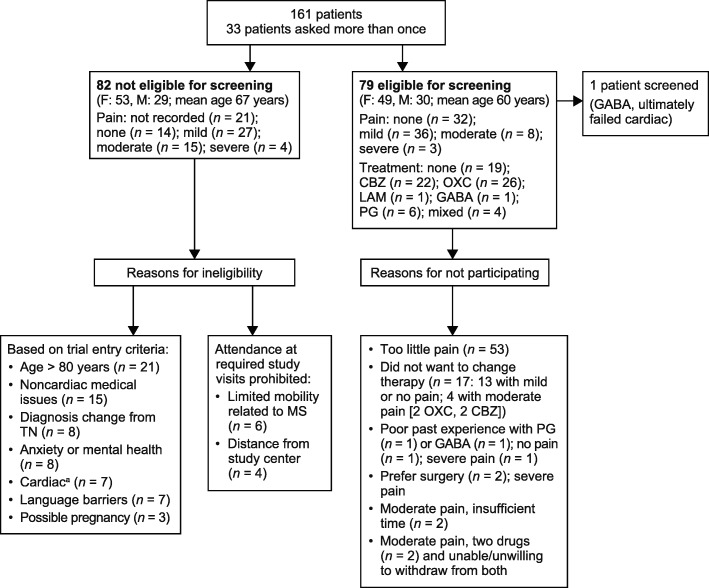
Detailed breakdown of prescreening and screening results from the London cohort. No pain = TN diagnosis but no current pain (on or off treatment); mild pain = score of 1–3; moderate pain = score of 4–7; severe pain = score of 7–10 [21]. ^a^Includes Fridericia’s formula requirements (< 450 ms in two of three electrocardiograms done at screening). Abbreviations: *CBZ* carbamazepine, *F* female, *GABA* gabapentin, *LAM* lamotrigine, *M* male, *MS* multiple sclerosis,* OXC* oxcarbazepine, *PG* pregabalin, *TN* trigeminal neuralgia

Figure 2 provides a more detailed breakdown of the prescreening and screening results for the London cohort, including reasons for noneligibility and screening failure. Of the eligible patients following prescreening, around two-thirds (53/79; 67.1%) had no or low-level pain at the time and were judged to not meet entry criteria for moderate pain and frequent daily paroxysms of pain. Around one-fifth (17/79; 21.5%) declined participation because they did not want to change from their current treatment regimen due to fear of losing pain control. Other reasons for not screening included the patient’s preference to receive surgery for severe pain, and reluctance to participate because of previous poor experience with pregabalin and gabapentin, the only permitted concomitant anticonvulsant medications.

At the Rome site, the majority of prescreened, eligible patients did not proceed to screening because of reluctance to change treatment regimen (50/84; 59.5%); other reasons were too little pain (4/84; 4.8%) and preference to receive surgery for severe pain (2/84; 2.4%). A detailed breakdown of the reasons for not screening among prescreened, eligible patients was not captured for the other sites, although the main issues experienced were similar to those in London and Rome.

**Fig. 3:**
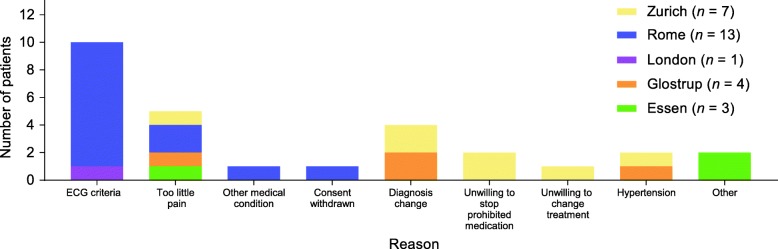
Reasons for failure among those screened at the five core sites. *ECG* electrocardiogram

Among those who were screened, reasons for failure also varied by site (Fig. 3).

### Analysis of open-label withdrawals

Across all 25 recruiting sites, 125 patients were screened, of whom 46.4% (58/125) did not meet study criteria for the open-label phase. A total of 67 patients were enrolled, of whom 44 completed the open-label phase and 29 entered the double-blind phase [16]. In order to further explore difficulties in retention even among individuals entering the trial, we reviewed the characteristics of individuals in the open-label population versus those completing the open-label phase and entering the double-blind phase (Table 2). Mean and median age were slightly higher for the open-label population than for patients entering the double-blind phase (mean 58.7 vs 55.3 years and median 60 vs 56 years, respectively). The proportion of females was similar between the open-label and double-blind populations (65.7 vs 65.5%, respectively). Height, weight, and body mass index were similar across groups.

**Table 2:** Demographic and clinical characteristics for patients entering the open-label versus double-blind phase^a^

Characteristic	Open-label phase (*n* = 67)	Double-blind phase (*n* = 29)
Age (years)		
Mean (SD)	58.7 (12.4)	55.3 (14.0)
Median (range)	60 (21–79)	56 (21–74)
Sex, *n* (%)		
Male	23 (34.3)	10 (34.5)
Female	44 (65.7)	19 (65.5)
Height (cm)		
Mean (SD)	167.3 (9.4)	167.3 (9.0)
Median (range)	165.0 (144–193)	165.0 (144–186)
Weight (kg)		
Mean (SD)	74.8 (14.3)	76.1 (13.4)
Median (range)	73.0 (47–107)	74.0 (55–107)
BMI (kg/m^2^)		
Mean (SD)	26.6 (3.9)	27.1 (3.8)
Median (range)	25.9 (19–35)	27.2 (22–33)
Race, *n* (%)		
White	64 (95.5)	28 (96.6)
African American/African heritage	1 (1.5)	1 (3.4)
Mixed race	2 (3.0)	0
TN duration (years), median (range)	6 (0–35)	6 (1–17)
Number of previous therapies for TN, median (range)	2 (1–25)	1 (1–6)
Anatomical site of TN pain, *n* (%)		
First branch	1 (2)	1 (3)
Second branch	17 (25)	9 (31)
Third branch	17 (25)	7 (24)
First and second branches	5 (8)	3 (10)
First, second, and third branches	7 (10)	2 (7)
Second and third branches	20 (30)	7 (24)

*BMI* body mass index, *SD* standard deviation, *TN* trigeminal neuralgia

^a^Full study population published in [16]

**Fig. 4:**
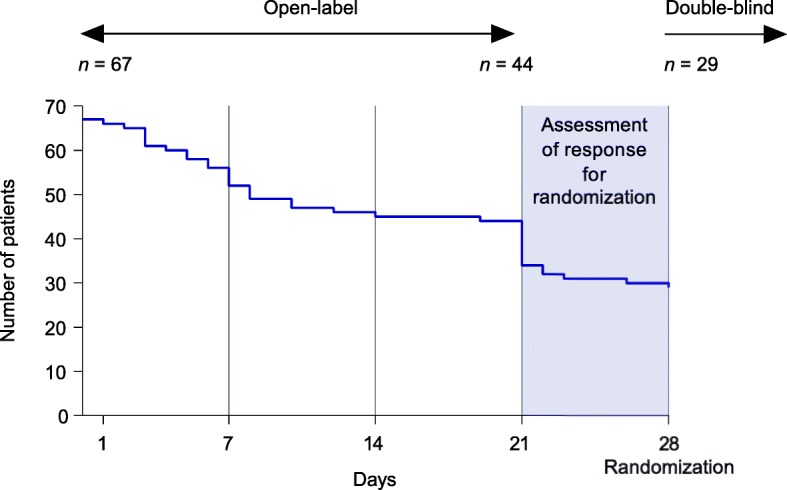
Withdrawals over time among patients recruited into the open-label phase (full study population)

We further assessed the timing and reasons for withdrawals during the open-label phase. A total of 23 patients withdrew before completing the open-label phase (day 21; Fig. 4), and approximately two-thirds of these (15/23; 65.2%) withdrew within the first 7 days. Among these patients, the documented reason for withdrawal was lack of efficacy for 18 (78.3%), withdrawal of consent for 2 (8.7%), and adverse events for 3 (13.0%). Of the three adverse events leading to withdrawal, two were considered probably related to the study drug (hypertension, decreased skin turgor, and dry mouth) and one was considered to be unrelated to the study drug (dyspnea).

## Discussion

Our analysis reinforces the challenges of patient recruitment in a rare disease clinical trial. Recruiting data from five countries enrolling patients for a phase IIa study showed that a high number of patients were noneligible at prescreening. The predominant reasons for ineligibility were diagnosis change, age > 80 years, and issues with language, distance, and mobility. Further barriers at screening included not meeting requirements for pain severity, reluctance to change medication, and failure on cardiac screening criteria. In addition, there were a high number of dropouts early in the open-label phase (Fig. 4), suggestive of potential anxiety after discontinuation of previous medications. It is clear from this analysis that a large number of patients must be reviewed in order to identify eligible patients in this population and provide a sufficient population to enroll a study population with enough power to inform scientific decision-making.

Diagnosis change was the most common reason for prescreening noneligibility. These patients had an initial diagnosis of classical TN, but on more careful phenotyping were found to have other types of facial pain (e.g., TN with concomitant pain, trigeminal autonomic cephalalgias, and multiple sclerosis). Older age was the second most frequent reason for noneligibility, despite the increase in the upper age limit to 80 years, indicative of the older demographic of the TN population. Many older patients had other complex medical problems that affected eligibility. Eligible patients were slightly younger than the noneligible group, which may be related to a greater probability of meeting exclusion criteria among older individuals. A number of patients were prescreening failures due to distance and mobility issues, including one-fifth of patients at the Glostrup site. The Glostrup site receives patients with TN from all over Denmark; hence, travel time could be up to 6 h. Some patients were also excluded because of language issues (e.g., migrants and patients from ethnic minority backgrounds).

Variations in screening and prescreening have several possible explanations. Each of the five countries that provided data for this analysis has a unique catchment and referral system that contributes to recruitment challenges. Centralized treatment accepts patients from a wide geographic region, thus patients who may need to travel large distances to get to the center and who may have issues with poor mobility may not be able to participate in a clinical trial. Conversely, centralized treatment may be of benefit in identifying patients eligible for a trial, especially in rare diseases. In addition, some centers chose not to perform the initial screening on patients known to have potential cardiac issues who required a substantial number of medications, accounting for the reasonably low number of noneligible patients at prescreening due to cardiac issues. Even so, at screening, a number of patients were excluded because of the regulatory requirements for ECG parameters (particularly at the Rome site), which may have led to more exclusions than expected due to the older age of the population.

Among the prescreened, eligible patients in London, a reasonable proportion did not meet requirements for pain severity; this may be because of changes in the pain phenotype over time since the initial diagnosis, the potential for remission [19], or good control on current therapies. Some patients with more severe pain opted to receive surgery instead of participating in the trial. A number of patients were also reluctant to stop their current anticonvulsants for fear of pain increase and/or the knowledge that they would need to return to their old medications after completion of the trial; this was a significant contributor to recruitment problems at the London site. These patients had also had poor experiences with gabapentin and pregabalin, which were the only drugs that were allowed. TN pain is unique compared with other chronic pain in terms of the intensity and unpredictability of attacks, which could increase such anxieties. In some cases, patients were unwilling to sacrifice their time/loss of income (e.g., for those who were self-employed) in order to attend multiple study visits. Nationally and internationally agreed criteria for diagnosis using published classifications could improve the initial phenotyping of patients.

The high number of withdrawals during the open-label phase of the trial, and particularly during the first few days, is suggestive of further barriers to trial retention. The documented reason for withdrawal for a majority of these patients was “lack of efficacy,” but precise reasons were not captured. It is possible that patients’ reluctance to proceed was related to anxiety after discontinuation of previous medications. Concomitant use of carbamazepine can decrease vixotrigine exposure, with effects shown to persist 7 days after discontinuation of carbamazepine [20], which may have contributed to perceived lack of efficacy early in the open-label phase (Fig. 4). Analysis of demographics among the open-label population and those entering the double-blind phase suggested that younger patients may have been slightly more likely to persist with the trial than older patients. Additionally, the use of a fixed dose with no potential for increase may have contributed to a lack of response. The ability to offer a long-term extension so responders could continue beyond the randomized controlled trial may have made a difference, as patients would not have had to go back on their old medication.

Recommendations for improving recruitment in future trials are summarized in Table 3. The vixotrigine phase III clinical trial is being designed with these recommendations in mind. The knowledge that the phase IIa study was successful and patient satisfaction was high will be helpful.

**Table 3:** Key recommendations for improving recruitment in trigeminal neuralgia (TN) clinical trials

Barrier (real or perceived)	Strategy
Patients with the opportunity to try study drug have to return to prior treatment at study completion	• Only true for phase II and earlier studies • Phase III studies should be designed with the option of a long-term extension
Required discontinuation of current therapy during the trial run-in phase	• Consider permitting use of and incorporating a higher/loading dose of the study drug to offset potential reduction in exposure owing to prior/concomitant medications • Implementation of a downtitration and uptitration algorithm of concomitant medications and study drug, respectively
Change in diagnosis from TN to other types of TN • The TN phenotype is also known to change over time, with potential for remission [22], which could alter whether patients meet pain requirements	• Recruitment of patients with recent visits to the clinic versus database records
Only specialists are recruiting patients attending specialist clinics	• Local primary care practices could be encouraged to telephone potential patients ○ A nurse could administer a semistructured questionnaire to identify potential cases for referral to the specialist center
Prescreening eligibility and screening failures inconsistently captured	• Implementation of a central project management and coordination system with prescreening and screening data locks that are promptly reviewed
Patient diary too large a burden	• Patient diaries less onerous (e.g., by not requiring documentation of all individual attacks, but rather average daily pain, or not requiring documentation of each attack after a certain threshold (e.g., > 20 attacks))
Lack of time or inability to attend study visits owing to distance/mobility	• Replace some onsite visits with telephone visits • Consider patient compensation for time loss and/or travel/dietary expenses

To improve recruitment, this study employed a randomized withdrawal design in order to minimize time on placebo, of particular importance in the TN population due to the severity of pain. However, many patients remained reluctant to come off their current therapy in order to participate. This was especially true for those individuals receiving > 1000 mg of carbamazepine or oxcarbazepine, because it is recommended that reductions are done slowly, and thus patients were fearful of pain worsening before the start of the trial. Further exploration of innovative designs that minimize the required sample size and/or maximize the number of patients receiving intervention could aid recruitment for future trials in TN and other rare diseases [5, 9, 17]. Open designs whereby patients know which treatment they are receiving have also been shown to improve recruitment, but they are subject to inherent bias [2].

Other factors identified in the broader literature with the potential to improve patients’ willingness to participate in clinical trials include good communication with patients [6, 7], commitment to share/make public trial results [7], dedicated research staff to support clinical staff and patients [8], ease of involvement (e.g., weekly vs daily diary, minimizing study visits) [6–8], the notion of contributing to medical knowledge and helping others [6, 8], higher satisfaction with care and physicians [7, 8], and limited anticipated side effects of a new drug [7]. Other strategies have also shown some success in improving recruitment, including telephone reminders to nonrespondents, and opt-out, rather than opt-in, procedures for contacting potential trial participants (although opt-out procedures are controversial as they may be perceived as not being truly voluntary) [2]. Direct-to-patient recruitment has been tried, utilizing a web-based approach to patient recruitment in which social media and foundation sites were used to reach out to patients directly, rather than through treatment centers [19]. Unfortunately, in this study, direct-to-patient recruitment was not as successful as recruitment through treatment centers and the authors suggest that further research using this method of recruitment is needed.

It is critical that investigators and authors evaluate and publicly share their recruiting strategies and difficulties when reporting clinical trials in order to inform other studies and support contextualization of data [4, 18].

These data must be interpreted with reasoned caution. This analysis of prescreening eligibility and screening failure was reliant on site logs; data were not formally databased for this study and complete records were not available for all recruiting sites. In addition, the reasons for noneligibility and screening failures may not have been captured consistently across sites.

## Conclusions

Recruitment challenges encompass not only motivating patients in participation and passing screening requirements, but also retaining them in the study once enrolled. A number of factors contributed to difficulties in recruitment in this phase IIa study in patients with TN, including diagnosis changes; issues relating to distance, language, and mobility; and anxieties over changing therapeutic regimen. Efforts should be made in future trials to overcome these barriers where possible. Centers with large numbers of patients are needed.

## Abbreviations

ECG: Electrocardiogram
TN: Trigeminal neuralgia
